# Spatial overlaps in the distribution of HIV/AIDS and malaria in Zimbabwe

**DOI:** 10.1186/s12879-018-3513-y

**Published:** 2018-11-27

**Authors:** Isaiah Gwitira, Amon Murwira, Joseph Mberikunashe, Mhosisi Masocha

**Affiliations:** 1 Department of Geography and Environmental Science, University of Zimbabwe, P. O. Box MP 167, Mount Pleasant, Harare, Zimbabwe; 2 Ministry of Health and Child Care, 4th Floor, Kaguvi Building, Central Avenue (between 4th and 5th Street), Harare, Zimbabwe

**Keywords:** Disease clusters, HIV/AIDS, Malaria, SaTscan, GIS, Spatial overlap

## Abstract

**Background:**

In most developing economies particularly in Africa, more people are likely to die of HIV/AIDS and malaria compared to other diseases. HIV/AIDS tends to be superimposed on the long standing malaria burden particularly in sub-Saharan Africa. The detection and understanding of spatial overlaps in disease occurrence is important for integrated and targeted disease control. Integrated disease control can enhance efficiency and cost-effectiveness through the development of drugs targeting multiple infections in the same geographic space.

**Methods:**

Using Zimbabwe as a case study, this study tests the hypothesis that malaria clusters coincide with HIV/AIDS clusters in space. Case data for the two diseases were obtained from the Ministry of Health and Child Care in Zimbabwe at district level via the District Health Information System (DHIS). Kulldorff’s spatial scan statistic was used to test for spatial overlaps in clusters of high cases of HIV/AIDS and malaria at district level. The spatial scan test was used to identify areas with higher cases of HIV/AIDS and malaria than would be expected under spatial randomness.

**Results:**

Results of this study indicate that primary clusters of HIV/AIDS and malaria were not spatially coincident in Zimbabwe. While no spatial overlaps were detected between primary clusters of the two diseases, spatial overlaps were detected among statistically significant secondary clusters of HIV/AIDS and malaria. Spatial overlaps between HIV/AIDS and malaria occurred in five districts in the northern and eastern regions of Zimbabwe. In addition, findings of this study indicate that HIV/AIDS is more widespread in Zimbabwe compared to malaria.

**Conclusions:**

The results of this study may therefore be used as a basis for spatially-targeted control of HIV/AIDS and malaria particularly in high disease burden areas. This is important as previous interventions have targeted the two diseases separately. Thus, targeted control could assist in resource allocation through prioritising areas in greatest need hence maximising the impact of disease control.

## Background

In most developing economies particularly in Africa, more people are likely to die of HIV/AIDS and malaria compared to other diseases. The two diseases remain the top killer infectious diseases in sub-Saharan Africa (SSA) [1]. It is estimated that SSA has more than 70% of the global HIV/AIDS burden and experiences 1.9 million new infections [2]. Globally, malaria remains one of the most serious public health problem associated with high morbidity and mortality [3]. An estimated 216 million cases were reported in 2016 with 90% of these cases occurring in the WHO African Region [4]. Since, HIV tends to be superimposed on the long standing malaria burden in sub-Saharan Africa [5, 6], we hypothesise that high malaria clusters should coincide with high HIV/AIDS clusters in space. This hypothesis is premised on previous research that has provided supporting evidence of biological interaction between HIV/AIDS and malaria at the patient level in most parts of Africa [7, 8]. While the transmission mechanisms of the two diseases differ, it has been reported that immune suppression by HIV/AIDS is associated with severe malaria [8–11]. The high health burden imposed by the two diseases on the economies of developing countries has increased public and private health spending with efforts aimed at reducing their impact. In 2016 alone, an estimated US$2.7 billion was invested in malaria control worldwide with 74% of this budget being used in Africa [4]. In this regard, understanding the spatial distribution of these two killer diseases is important for enhancing effective control since knowledge of geographic distribution of disease burden is important for targeted intervention.

Within Africa, the burden of malaria varies from country to country. In Zimbabwe, malaria remains a major public health threat. For instance, over half of the total human population of 13.5 million remain at risk of contracting the disease [12]. Although deaths due to HIV/AIDS and malaria have drastically declined as a result of international support particularly from the Global Fund, the two diseases remain the top causes of mortality and morbidity in Zimbabwe [1]. Several studies have shown an increase in co-infection of HIV/AIDS and malaria across the globe. These studies have revealed that HIV/AIDS and malaria may interact bilaterally with HIV/AIDS altering the natural history of malaria and vice versa [10, 13]. For example, [14] showed that HIV/AIDS prevalence tends to be higher among patients with severe malaria than in the general population in Bangladesh. A related study by [15] conducted in Zimbabwe found that HIV/AIDS-infected adults were more likely to develop malaria complications than uninfected adults and were more than twice likely to die. In addition, [16] found that HIV/AIDS prevalence in adults was 5–6% higher in adults with severe malaria in Burkina Faso. Work done in Kenya also documented that HIV/AIDS and malaria co-infection may be responsible for the increase in HIV/AIDS infections and malaria episodes [5]. In fact, it is estimated that the interaction of malaria and HIV/AIDS in a district in Kenya could have resulted in 980,000 excess malaria cases and as much as 8500 HIV/AIDS excess infections in 2009 alone [8]. Overall, these studies suggest malaria can facilitate the spread of HIV/AIDS and hasten mortality [5, 17].

Although, previous research has demonstrated that HIV/AIDS and malaria co-infection is highly likely, much emphasis has been placed on co-infection and co-morbidity at the patient level [18–20]. While co-infection and co-morbidity are clinically and epidemiologically important [21, 22], understanding overlap of the two diseases in space is important for integrated disease control and management as well as targeted control [21] and yet few studies have focussed on co-distribution. While some studies done in different countries such as Malawi, Mozambique, Tanzania, Kenya, Zambia, Lesotho and Zimbabwe [18, 19] show that malaria and HIV/AIDS have an overlapping geographic distribution at a country scale, a gap still exists with regard to identifying specific regions of spatial overlap of the two diseases at finer scales. Closing this gap may pave way for more research to determine whether the disease overlaps translate into co-infection at the patient scale [13, 23]. This is particularly important given the fact that HIV/AIDS and malaria interact bidirectionally and synergistically with each other [9–11]. This explains the observation that infection with HIV/AIDS tends to increase the risk and severity of malaria infection [7, 13, 24]. Therefore, development and adoption of spatial techniques capable of detecting spatial disease overlaps is critical to inform targeted control as well as integrated management. Integrated disease control can enhance efficiency and cost-effectiveness through the development of drugs for targeting multiple infections [21, 25].

One of the promising techniques to detect spatial overlaps in disease occurrence is based on use of spatial scan statistics. For example spatial scan statistics was used to map HIV/AIDS clustering and spatial variability based on sampled data from Demographic and Health Surveys (DHS) in several sub-Saharan countries including Zimbabwe [19, 20]. The results showed that the spatial distribution of clusters of high and low HIV/AIDS prevalence vary in space. Recently, [26] detected spatial hotspots of malaria in different parts of Zimbabwe and found that malaria hotspots occur in the south east, east and north east of the country. None of the previous studies [18, 27] investigated and mapped co-distribution of HIV/AIDS and malaria spatial clusters despite the importance of this information to planning large-scale integrated and targeted disease control programmes [14, 28]. Using Zimbabwe as a case study, this study tests the hypothesis that malaria clusters coincide with HIV/AIDS clusters in space since HIV/AIDS tends to be superimposed on the long standing malaria burden in sub-Saharan Africa [6, 29].

Although several studies indicate a close link between HIV/AIDS and TB as a result of the biological explanation for their co-existence [7, 8, 10, 30], recently the co-existence of HIV/AIDS and malaria has received more attention [9]. Researchers have indicated evidence of biological interaction between HIV/AIDS and malaria whose combined impact on health systems has been observed to be substantial in most parts in Africa [7, 8, 10, 30]. The rationale for this study therefore was to test for the existence of spatial overlaps in the geographic distribution of HIV/AIDS and malaria which could provide an indication of the disease burden in a region. The existence of spatial overlaps may then be used as the basis for further research to explore whether the existence of spatial overlaps at population level also translates into co-infection by the two diseases at the patient scale.

## Methods

### Study area

The study was carried out in Zimbabwe with a population of about 13.5 million based on the 2012 national census. The country is located in southern Africa between latitudes 15° 30″ and 22° 30″ S and longitudes 25° 00″ and 33° 10″ E. Its total area is 390,757 km^2^. The country is dominated by communal land ownership interspaced with commercial farming land. Zimbabwe’s climate is subtropical with distinct seasons moderated by altitude resulting in relatively humid conditions at high altitudes [31, 32]. The altitude of the study area ranges from less than 300 m to 2590 m above mean sea level. Mean annual rainfall ranges from below 400 mm in the southern and north-western parts to over 1000 mm in the eastern and central parts of the country. The country records its lowest minimum temperatures in June or July while the maximum temperatures are experienced in October. Mean monthly temperatures vary from 15 °C in July to 24 °C in November, while the mean annual temperature varies from 18 °C in the high-altitude areas to 23 °C in the low altitude areas.

### Data sources

Data for positive malaria cases used in this study were downloaded from the District Health Information Software (DHIS 2) with the authority of the Ministry of Health and Child Care (MOHCC) in Zimbabwe. The annual positive malaria cases used in this study were recorded at 1300 geocoded health facilities distributed throughout Zimbabwe in 2016. The data were then aggregated by district in a geographic information system (GIS). The health facilities where malaria cases were recorded included government clinics, hospitals, rural health centres and rural district council clinics. In Zimbabwe malaria is a notifiable disease. Hence, screening for the disease is done when a suspected individual visits a health centre. The screening and testing for malaria is done using rapid diagnostic tests or microscopy. This is carried out by trained health personnel [33]. After the data is recorded at health facility level, it is then aggregated into district totals and entered into the DHIS2.

The HIV/AIDS case data were obtained from the HIV/AIDS and TB unit housed under the Ministry of Health and Child Care in Zimbabwe. The data were obtained from HIV/AIDS testing centres located at various health facilities within the districts in the country. The HIV/AIDS data were for all diagnosed cases and not necessarily newly diagnosed cases. The data were only for patients tested at health facilities in a particular district. The dataset did not indicate whether a patient who moved from another district will have to be re-tested. Thus, a patient was recorded in a district where they have been tested regardless of that district being their home district. The data used in this study were for all annual HIV/AIDS cases in 2016 for patients who presented themselves for medical care regardless of whether they were retained for medical care or not.

In total, HIV/AIDS and malaria data were obtained from 71 administrative districts based for the year 2016. The HIV/AIDS and malaria prevalence per district was calculated by dividing the total number of disease cases recorded in each district by the total human population of that district. The resultant ratio was then multiplied by 1000 to standardise the prevalence so that they reflect disease prevalence per 1000 persons in a population which is easy to interpret. The rationale for using aggregated district level malaria and HIV/AIDS data is that disease interventions in Zimbabwe are planned and implemented using the administrative district as the spatial epidemiological unit [34].

### Population data

In addition to HIV/AIDS and malaria case data, population data for the target districts were also used in this study. The population data were obtained from the Zimbabwe Statistical Office (ZimStat) at district level. The total population for each district was estimated based on a projected annual growth rate of 1.2% from the 2012 National population census [35]. The 2012 data was used for estimating the population for 2016 as it is not a census year [36]. In Zimbabwe, the population of intercensal years is estimated from projected annual growth rates from the period of the latest census.

### Statistical analysis

Significant spatial clusters of high HIV/AIDS and malaria cases were detected in each district by applying Kulldorff’s spatial scan statistics [37]. This technique has been widely used to detect clusters in many different applications [37–41]. In its several applications, the technique has been found to have reasonable sensitivity and specificity which enhances its efficiency and accuracy compared to other cluster detection techniques such Bayesian disease mapping [39, 42, 43]. The detection of spatial clusters of HIV/AIDS and malaria cases was carried out based on the Poisson probability model. The main assumption of the Poisson probability model is that HIV/AIDS and malaria cases in each district follow a Poisson distribution meaning that the disease cases resulted from a random process according to a known underlying population at risk [19].

In this study, three sets of data were built for the analysis of both HIV/AIDS and malaria cases based on discrete Poisson model in SaTScan software version 9.4.2. The datasets were: a case file representing separately the annual number of cases for both HIV/AIDS and malaria for each district (*n* = 71) for 2016; a coordinate file representing geographic coordinates of the centroid of each district polygon; and a population file representing the projected total population for each district in 2016. The centroid of each district was calculated in a GIS environment. The spatial join function was used to link disease cases to the centroid of the districts. The disease cases were then converted to SaTscan format for use in the detection of spatial clusters. The statistically significant clusters of high HIV/AIDS and malaria cases were evaluated using a circular window [43]. The circular window was centred on each district centroid. The radius was of the circular window was up to a maximum value not exceeding 50% of the population at risk. The default 50% of the population at risk was adopted here as it is widely used in the literature [43–45]. In addition, the selection of the default 50% of the population at risk as the maximum radius for detecting clusters is also based on the premise that for a relatively large country such as Zimbabwe, at a national scale the impact of scan radius on the results is negligible [46]. Hence, we adopted the recommended default settings as these settings are known to yield optimal clusters reflecting the true underlying disease pattern [43].

To test the statistical significance of each detected cluster of HIV/AIDS or malaria cases, a Poisson generalized log likelihood ratio was calculated based on Monte-Carlo inference procedure [47]. In addition, to ensure adequate statistical power in detecting clusters, 999 Monte Carlo replications were performed under the null hypothesis of random distribution of either HIV/AIDS or malaria cases [47–49]. The observed and expected HIV/AIDS or malaria cases within and outside the circular window were compared to the log likelihood ratio. Formally, the likelihood function was calculated as:

1 $$ {LR}_{(z)}={\left(\frac{C}{E_{\left[c\right]}}\right)}^c{\left(\frac{C-c}{C-{E}_{\left[c\right]}\ }\right)}^{C-c}I\left(\frac{c}{E_{\left[c\right]}}>\frac{C-c}{C-{E}_{\left[c\right]}\ }\right) $$

where *C* is the total number of HIV/AIDS or malaria cases in the study area, *c* is the observed number of HIV/AIDS or malaria cases within the window, and *E*_[*c*]_ is the covariate adjusted expected number of cases within the window under the null hypothesis. Since the analysis is conditioned on the total number of cases observed, *C*-*E*_[c]_ is the expected number of HIV/AIDS or malaria cases outside the window. $I\left(\frac{c}{E_{\left[c\right]}}>\frac{C-c}{C-{E}_{\left[c\right]}\ }\right)$ is an indicator function used when scanning for clusters with high cases of HIV/AIDS or malaria.

Based on the log likelihood ratio test, the cluster with the maximum log likelihood ratio was defined as the most likely cluster (herein referred to a primary cluster) while other clusters with statistically significant but low log likelihood values were defined as secondary clusters [48]. Statistical significance of clusters was determined through Monte Carlo simulations. The criterion of ‘no geographical overlap’ was used to report secondary clusters. In the results section, the primary and secondary clusters are presented in separate maps to avoid clutter. For each detected primary or secondary cluster, the strength of clustering was characterised using the relative risk (RR). RR was calculated as the ratio of the observed number of cases of HIV/AIDS or malaria cases within each cluster to the number of cases outside the cluster. In this case, high RR indicates strong clustering of a given disease. The detected clusters were exported to ArcGIS version 9.3 [50] for visualisation and cartographic displays.

## Results

### Malaria and HIV/AIDS prevalence in Zimbabwe

**Fig. 1:**
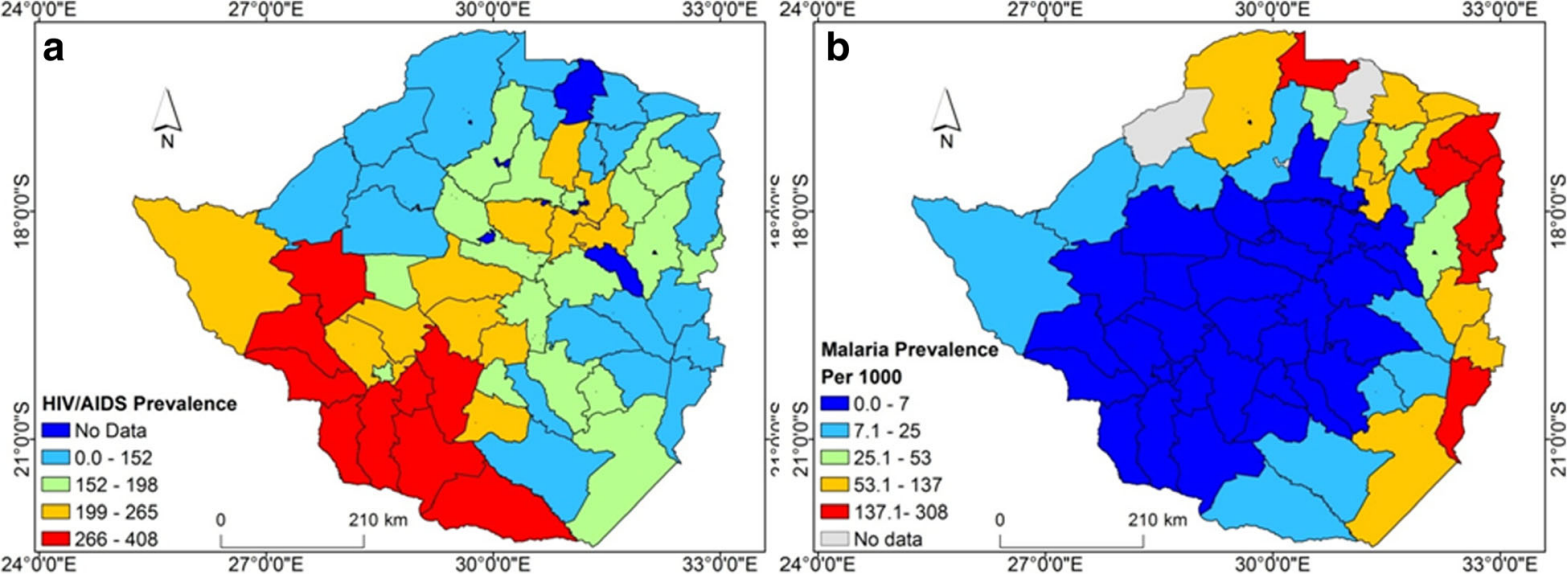
Spatial distribution of annual (**a**) HIV/AIDS and (**b**) Malaria prevalence per 1000 of the population in Zimbabwe in 2016

Figure 1 illustrates HIV/AIDS and malaria prevalence at district scale in Zimbabwe. High HIV/AIDS prevalence was observed in the south, south-west and central regions of the country while the eastern and northern regions of the country are characterised by low prevalence (Fig. 1a). In contrast, high malaria prevalence was observed in the eastern, north-eastern and northern regions of the country (Fig. 1b).

### Spatial clusters of HIV/AIDS and malaria

**Fig. 2:**
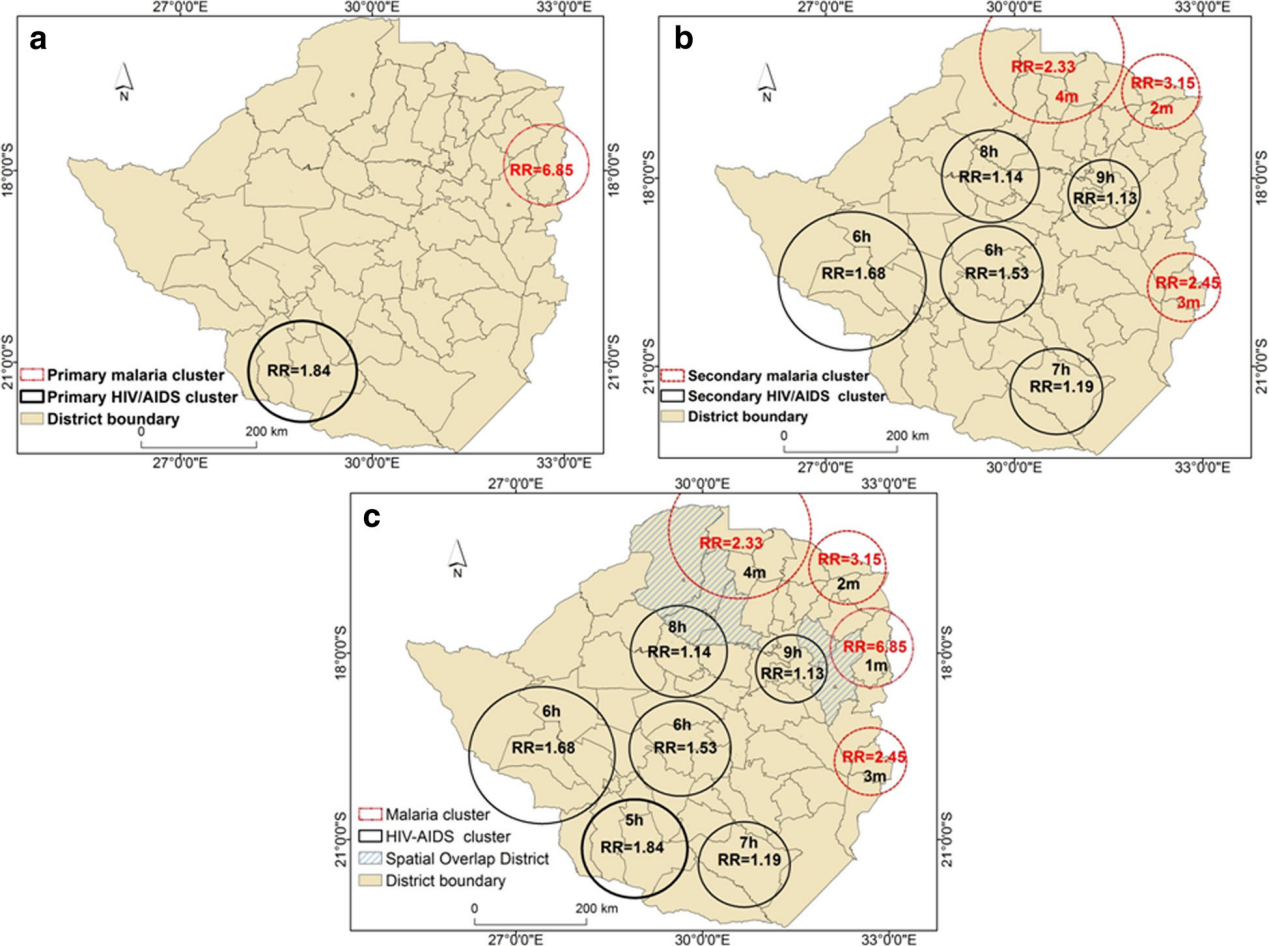
Distribution of HIV/AIDS and malaria primary and secondary clusters and their relative risk in Zimbabwe. *Black Circles indicate HIV/AIDS clusters while the red circles indicate malaria clusters. Districts with hatching pattern indicate areas in which HIV/AIDS and malaria overlap in space. The letters h and m on the cluster numbers indicate HIV/AIDS and malaria clusters, respectively. RR denotes reletive risk*

Figure 2 illustrates the location of statistically significant spatial clusters of high HIV/AIDS and malaria cases in Zimbabwe. The significant primary cluster for HIV/AIDS with a RR of 1.84 (*p* < 0.05) was detected in the southern region of the country. This primary HIV/AIDS cluster overlapped with seven districts namely Beitbridge, Gwanda, Matobo, Mangwe, Insiza, Umzingwane and Mwenezi (Fig. 2a). Whereas the primary HIV/AIDS cluster was located in the southern region, the primary cluster of malaria occurred in the north-eastern region of the country (Fig. 2a). This primary significant (*p* < 0.05) malaria cluster with a RR = 6.85 overlapped with five districts of Mutasa, Nyanga, Murewa, Mudzi, Makoni. From the results in Fig. 2a, one observes that the primary HIV/AIDS and malaria clusters had no spatial overlap.

In addition to the primary HIV/AIDS cluster, five secondary clusters, which overlapped with 31 districts situated in the southern and central regions of the country were detected (Fig. 2b). The RR for the secondary HIV/AIDS clusters ranged from 1.13 to 1.68. The results in Fig. 2b also illustrate the distribution of three secondary malaria clusters. These clusters were concentrated in the northern and eastern regions of Zimbabwe and overlapped with 19 distrcicts. The RR of the secondary malaria clusters ranged from 2.33 to 3.15.

Results of this study also illustrate that there were five districts where spatial overlap in the two diseases occurred (Fig. 2c). Out of the five districts, two districts detected as HIV/AIDS secondary clusters overlapped with a primary malaria cluster. The other three districts had secondary clusters for both HIV/AIDS and malaria overlapping with each other (Fig. 2c). Spatial overlap of HIV/AIDS and malaria occurred in the districts of Makonde, Zvimba, Hurungwe, Murewa and Makoni. The general description of the clusters of high HIV/AIDS and malaria cases is provided in Table 1. The table illustrates the primary as well as secondary HIV/AIDS and malaria clusters, the number of observed cases and the corresponding expected number of cases based on purely spatial scan statistic. Overall, the primary and secondary HIV/AIDS and malaria clusters were statistically significant (*p* < 0.05). In addition, the most likely clusters for HIV/AIDS and malaria had higher than expected cases (Table 1).

**Table 1:** Statistically significant clusters of HIV/AIDS and malaria as well as primary and secondary clusters

Cluster ID	Radius (km)	LLR	Observed	Expected	*P* value
^a^1m	70	45,502	45,486	7785	0.001
2 m	65	11,784	26,777	9131	0.001
3 m	122	8448	33,149	15,289	0.001
4 m	60	7446	26,103	11,334	0.001
^a^5h	87	9566	64,063	35,511	0.001
6 h	122	9214	84,885	51,891	0.001
7 h	85	6910	91,935	61,474	0.001
8 h	60	1318	70,378	57,902	0.001
9 h	76	428	31,141	26,306	0.001
10 h	81	300	39,216	34,624	0.001

^a^denotes primary cluster with the highest Log Likelihood ratio, (1 m–4 m: Malaria clusters and 5 h–10 h: HIV/AIDS clusters)

## Discussion

This study tested the hypothesis that in environmental settings that favour malaria and HIV/AIDS, clusters of HIV/AIDS coincide with clusters of malaria. This was premised on the understanding that a synergetic relationship exists between malaria and HIV/AIDS in which the prevalence of HIV/AIDS tends to be higher among patients with severe malaria during co-infection [14, 15]. Results of this study indicate that the primary clusters of HIV/AIDS and malaria were not spatially coincident across Zimbabwe. The HIV/AIDS primary cluster was located in the southern districts of Beitbridge, Gwanda, Matobo, Mangwe, Insiza, Umzingwane and Mwenezi whereas the primary cluster for malaria was located in the eastern and northern districts of Mutasa, Nyanga, Murewa, Mudzi, Makoni. While spatial overlaps were not detected when primary clusters were considered, the results of this study uncovered spatial overlaps in the distribution of statistically significant secondary clusters of HIV/AIDS and malaria. Spatial overlap in the distribution of secondary clusters HIV/AIDS and malaria was observed in three northern districts of Makonde, Zvimba, and Hurungwe. In addition, two districts of Murewa and Makoni located in the northern and eastern regions of the country were characterised as having a significant HIV/AIDS secondary cluster that spatially coincided with a primary malaria cluster.

The findings of this Zimbabwean study shed insight into the spatial variation in the pattern of malaria and HIV/AIDS burden since both diseases were analysed simultaneously. An important feature of the results is that there are some districts characterised by the co-distribution of HIV/AIDS and malaria clusters whereas others are dominated by clusters of either disease. This is the first time overlaps between malaria and HIV/AIDS are being reported at the spatial epidemiological scale at which interventions are undertaken. In this regard, the findings of the study provide entry points as to which districts should receive priority attention in the targeted treatment of the two diseases. This is particularly important for resource limited countries that rely on external funding to meet their health obligations. Previous interventions tended to target the diseases separately which is not cost effective [51]. Strategies for malaria and HIV/AIDS integrated control might include mounting simultaneous interventions targeting the same community in a geographic space including awareness campaigns, sex education, use of insecticide treated nets and improving their living conditions. Such an approach offers the potential to share fixed costs when reaching out to the same community [24, 51]. Lack of evidence of spatial overlaps in disease distribution has been a major hindrance to the introduction of integrated disease control [52, 53]. Previously, studies of this nature have been used to guide integrated control of *Schistosomiasis mansoni* and the Soil transmitted helminths in East Africa [21].

Considering that the socio-economic environment that favours HIV transmission can coincide with the environmental template in which malaria transmission occurs [26, 54], HIV occurrence is likely modified by endemicity and stability of malaria transmission [23, 55]. Previous work has shown that in areas characterised by stable malaria transmission, HIV/AIDS may increase the risk of infection by malaria particularly in adults with advanced immuno-suppression [23, 51, 55]. In addition, in areas characterised by unstable malaria transmission, there is increased risk of complicated and severe malaria deaths among HIV/AIDS-infected adults due to suppressed natural immunity [30, 56]. Moreover, in areas of high-intensity malaria transmission, HIV/AIDS has been found to increase the incidence of clinical malaria among adults [56].

The mechanisms to explain co-distribution of HIV/AIDS and malaria were beyond the scope of this study. However, previous work found that at the patient level malaria is likely to facilitate co-infection in HIV/AIDS-infected individuals living in areas with high malaria prevalence [57]. Findings from a study in Zimbabwe revealed that malaria symptoms were likely to develop four-fold in HIV/AIDS-infected pregnant women than in those without HIV/AIDS [58]. In fact, HIV/AIDS has been found to increase malaria parasite density which is an indicator of malaria severity [59]. A recent poverty assessment survey in Zimbabwe revealed that the five districts with spatial overlap of HIV/AIDS and malaria had a high poverty prevalence rate ranging from 61 to 89% [60]. The average poverty prevalence of these districts was 75%, which is higher than the average national poverty prevalence of 63% [60]. Poverty in this study was defined as the welfare level of a population [60]. It was derived by considering multiple indicators such as housing quality, level of education and income levels that characterise the social and economic performance of communities [60]. Although the influence of poverty on HIV/AIDS and malaria prevalence was not formally tested in this study, the existence of spatial overlaps in the five poor districts implies that the high HIV/AIDS burden is superimposed on a suitable environment for malaria. In fact spatial overlap between malaria and HIV/AIDS may result from the fact that both are diseases of poverty often affecting the poorest communities in a population lacking access to education, information and state services [19].

The results of the current study justify the need for spatially-targeted control and prevention strategies for HIV/AIDS and malaria in Zimbabwe. Targeted control and management of the two diseases particularly in areas of high disease burden assists in resource allocation through prioritising areas in greatest need hence maximising the impact of disease control [43, 49, 61]. Although HIV/AIDS and malaria have different transmission mechanisms and treatment regimes, in order to reduce the lethal consequences of dual infection, prevention and treatment programmes may mutually reinforce each other [7, 9]. This may be made possible by the immense potential for synergism in the treatment of the two diseases [30]. The results of this study indicating that in five districts the high HIV/AIDS and malaria burdens are spatially coincident imply that the population in those districts tend to be more vulnerable to malaria. As such, the provision of insecticide-treated nets should be a high priority [62]. In fact malaria prevention through vector control using insecticide treated nets and residual spraying has been shown to be an effective strategy to curb HIV prevalence [7]. For example, in Kenya the provision of long-lasting insecticide-treated nets to HIV/AIDS infected patients was observed to result in a significant reduction in the progression of HIV/AIDS [63]. In this case, the control of malaria becomes a practical and cost-saving method in resource-limited rural areas of sub-Saharan Africa [7].

In addition, results of this study could be useful in the design of programmes for integrated management of the HIV/AIDS and malaria. This calls for collaborations between health authorities, the research community and local government officials to ensure integrated service delivery through the provision of better diagnostic tools for both diseases [62]. In addition, spatially explicit information on the occurrence of HIV/AIDS and malaria clusters may be used to strengthen the health delivery system using an integrated approach [20]. Thus, interventions such as education and awareness campaigns, prevention, treatment and care, and optimum location of referral health centres may be guided by the mapping of HIV/AIDS and malaria clusters.

Comparatively, the findings of this study indicate that HIV/AIDS is more widespread in Zimbabwe. In addition to the large primary HIV/AIDS cluster, this study detected a total of five significant secondary HIV/AIDS clusters, which was one -and-half times more than the number of malaria clusters. The five secondary clusters, overlapped with a total of 31 districts situated in the southern and central regions of the country whereas the three significant secondary malaria clusters overlapped with 19 districts. The HIV/AIDS clusters were mostly distributed in the southern and central regions of the country. This suggests that the social and behavioural factors such as heterogeneity in epidemic phases or changes in sexual risk behaviour, or uptake of prevention and treatment interventions and migration patterns of high-risk individuals are more prevalent in these regions as reported in a previous regional study [20]. Although the results are in line with findings of previous studies, there is need for further research to understand factors that can explain the wide geographic range of HIV/AIDS in Zimbabwe.

The localised pattern of malaria clusters suggests strong environmental control of the disease. The eastern and northern regions are characterised by high suitability of two main malaria vectors that is, *Anopheles arabiensis* and *Anopheles funestus* [26]. The two regions are also characterised by low altitude, ideal temperature and rainfall which provide suitable conditions for malaria transmission in addition to insecticide resistance [64–66]. Although the results are in line with several studies that classify malaria as an environmental disease, the pattern also reflect the impact of malaria control interventions in reducing the disease burden. This may be supported by previous studies which showed that although the southern region have suitable environmental conditions for malaria transmission, most districts in this region are in the malaria pre-elimination phase resulting in the observed low prevalence [12, 67, 68].

What makes this study different from previous studies is in modelling the spatial co-distribution of HIV/AIDS and malaria. Previous studies analysed spatial clustering of these two diseases in isolation despite their potential spatial co-occurrence. Modelling the spatial co-occurrence of HIV/AIDS and malaria provides more insight for possible spatial overlap in the distribution of the two diseases which is absent when the diseases are considered in isolation. The approach adopted in this study makes a useful contribution to spatial epidemiology since clinical results have indicated that there is a high possibility of co-infection of the two diseases resulting from their close association [18–20].

A limitation that could have affected the results of this study is that the georeferenced disease incidence data used in this analysis was available at a coarse scale (district level) which tends to mask micro-geographic variations in disease prevalence. Results of this study could be improved by using disease incidence at health facility level. Another limitation of this study is that in the few districts where HIV/AIDS and malaria clusters overlapped, it is not known whether individual persons in these regions are co-infected and yet this information is useful to guide control strategies. Although previous studies have indicated that in the presence of HIV/AIDS, patients tend to suffer from severe malaria, we used malaria prevalence data as reliable patient level data from health information systems was not available. The limitation of malaria prevalence is that it is affected by the population of the district. Despite these limitations, the findings of this Zimbabwean study highlight the advantages of harnessing geospatial technologies for improved disease mapping and surveillance for informed disease control.

## Conclusion

This study investigated whether there is spatial congruence in the distribution of two diseases of global public health importance that is HIV/AIDS and malaria. Using geo-referenced prevalence data reported at district level in Zimbabwe and a widely used spatial clustering technique, the study successfully detected spatial overlaps in the distribution of HIV/AIDS and malaria in five northern and eastern districts of Zimbabwe. Although the spatial overlap was limited in space, this is the first time this result is being reported in Zimbabwe. This key finding opens new opportunities for geographically-targeted integrated intervention strategies for HIV/AIDS and malaria in Zimbabwe as well as other tropical countries with similar geographical settings. In addition, this study opens up opportunities for field studies in districts with spatial overlap to test whether there is also co-infection.

## Abbreviations

AIDS: Acquired Immune Deficiency Syndrome
HIV: Human Immunodeficiency Virus
WHO: World Health Organisation
ZIMSTAT: Zimbabwe National Statistics

## References

[1] Barley KD, Murillo S, Roudenko AM. Tameru, Tatum S. A mathematical model of HIV and malaria co-infection in sub-Saharan Africa*.* Journal of AIDS Clinic Research 2012;3(173):doi:10.4172/2155-6113.1000173.

[2] Kharsany ABM, Karim QA (2016). HIV infection and AIDS in sub-Saharan Africa: current status, challenges and opportunities. Open AIDS Journal.

[3] Dewald JR, Fuller DO, Müller GC, Beier JC (2016). A novel method for mapping village-scale outdoor resting microhabitats of the primary African malaria vector, Anopheles gambiae. Malar J.

[4] World Health Organization (WHO) (2017). World Malaria Report 2017.

[5] Abu-Raddad LJ, Patnaik P, Kublin JG (2006). Dual infection with HIV and malaria fuels the spread of both diseases in sub-Saharan Africa. Science.

[6] UNICEF. Strategic framework for the Prev Control of HIV/AIDS and STI’s Within Somali Population. Nairobi: English Press Limited; 2003.

[7] Kwenti TE (2018). Malaria and HIV coinfection in sub-Saharan Africa: prevalence, impact, and treatment strategies. Research and Reports in Tropical Medicine.

[8] Reithinger RMR, Kamya CJM, Whitty G, Dorsey VSH. Interaction of malaria and HIV in Africa. BMJ. 2009. 10.1136/bmj.b214. 10.1136/bmj.b2141 19493941

[9] Franke MF, Spiegelman D, Ezeamama A (2010). Malaria parasitaemia and CD4 T cell count, viral load and adverse HIV outcomes among HIV-infected pregnant women in Tanzania. Am J Trop Med Hyg.

[10] Nyabadza F., Bekele B. T., Rúa M. A., Malonza D. M., Chiduku N., Kgosimore M. (2015). The Implications of HIV Treatment on the HIV-Malaria Coinfection Dynamics: A Modeling Perspective. BioMed Research International.

[11] David AM, Mercado SP, Becker D, Edmundo K, Mugisha F (2007). The prevention and control of HIV/AIDS, TB and vector-borne diseases in informal settlements: challenges, opportunities and insights. J Urban Health.

[12] Manyangadze T, Chimbari MJ, Macherera M, Mukaratirwa S. Micro-spatial distribution of malaria cases and control strategies at ward level in Gwanda district, Matabeleland SouthZimbabwe. Malaria Journal. 2017;16(476). 10.1186/s12936-017-2116. 10.1186/s12936-017-2116-1 PMC5697109 29162102

[13] Alemu A, Shiferaw Y, Addis Z, Mathewos B, Birhan W (2013). Effect of malaria on HIV/AIDS transmission and progression. Parasit Vectors.

[14] Bastos FI, Barcellos C, Lowndes SM, Friedman SR (1999). Co-infection with malaria and HIV in injecting drug users in Brazil: a new challenge to public health. Addiction.

[15] Chirenda J, Siziya S, Tshimanga M (2000). Association of HIV infection with the development of severe and complicated malaria cases at a rural hospital in Zimbabwe. Cent Afr J Med.

[16] Lagarde E, Congo Z, Meda N, Baya B, Yaro S, Sangli G (2004). Epidemiology of HIV infection in urban Burkina Faso. Int J STD AIDS.

[17] Herrero MD, Rivas P, Rallon N, Ramirez-Olivencia G, Puente S (2007). HIV and malaria. AIDS Rev.

[18] Alemu K, Worku A, Berhane Y (2013). Malaria infection has spatial, temporal, and spatiotemporal heterogeneity in unstable malaria transmission areas in Northwest Ethiopia. PLoS One.

[19] Cuadros DF, Awad SF, Abu-Raddad LJ (2013). Mapping HIV clustering: a strategy for identifying populations at high risk of HIV infection in sub-Saharan Africa. Int J Health Geogr.

[20] Cuadros DF, Abu-Raddad LJ (2014). Spatial variability in HIV prevalence declines in several countries in sub-Saharan Africa. Health &Place.

[21] Clements ACA, Marie-Alice D, Ndayishimiye O, Brooker S, Fenwick A (2010). Spatial co-distribution of neglected tropical diseases in the east African Great Lakes region: revisiting the justification for integrated control. Trop Med Int Health.

[22] Pullan RL, Sturrock HJW, Magalhaes RJS, Clements ACA, Brooker SJ (2012). Spatial parasite ecology and epidemiology: a review of methods and applications. Parasitology.

[23] World Health Organization (WHO) (2004). Technical Consultation on Malaria and HIV and their Interactions and Public Health Policy Implications.

[24] Wumba RD, Zanga J, Aloni MN, Mbanzulu K, Kahindo A, Mandina MN, Ekila MB, Mouri O, Kendjo E (2015). Interactions between malaria and HIV infections in pregnant women: a first report of the magnitude, clinical and laboratory features, and predictive factors in Kinshasa the Democratic Republic of Congo. Malaria Journal.

[25] World Health Organisation (WHO) (2007). Report of the first meeting of WHO Strategic and Technical Advisory Group on Neglected Tropical Diseases.

[26] Gwitira I, Murwira A, Zengeya FM, Shekede MD (2018). Application of GIS to predict malaria hotspots based on Anopheles arabiensis habitat suitability in southern Africa. Int J Appl Earth Obs Geoinformation.

[27] Wiwatnitkit V (2006). Co-infection between TB and malaria: a consideration on interaction of molecules and pathogenesis. Journal of vector-borne diseases.

[28] Kulldorff M, Nagarwalla N (1995). Spatial disease clusters: detection and inference. Stat Med.

[29] Adjuik M, Smith T, Clark S, Todd J, Gamb A, Kinfu Y, Kahn K, Mola M (2006). Cause specific mortality rates in sub-Saharan Africa and Bangladesh. Bulletin of World Health Organisation.

[30] Cuadros DF, Branscum AJ, Crowley PH (2011). HIV-malaria co-infection: effects of malaria on the prevalence of HIV in east sub-Saharan Africa. Int J Epidemiol.

[31] Marshall B (2010). The fishes of Zimbabwe and their biology.

[32] Torrance JD (1981). Climate handbook of Zimbabwe.

[33] Ministry of Health and Child Care (MOHCC) (2014). Zimbabwe National Malaria Control Programme 2014–2015.

[34] Midzi N, Kavhu B, Manangazira P, Phiri I, Mutambu SL, Tshuma C, Chimbari MJ, Munyati S, Midzi SM, Charimari L, Ncube A, Mutsaka-Makuvaza MJ, Soko W, Madzima E, Hlerema G, Mbedzi J, Mhlanga G, Masocha M. Inclusion of edaphic predictors for enhancement of models to determine distribution of soil-transmitted helminths: the case of Zimbabwe. Parasites & Vectors. 2018;11(47):1–13. 10.1186/s13071-017-2586-6 PMC5775612 29351762

[35] Zimbabwe National Statistics Agency (ZIMSTAT) (2012). Zimbabwe Population Census 2012.

[36] Zimbabwe National Statistics Agency (ZIMSTAT) (2015). Zimbabwe Population Projections Thematic Report.

[37] Kulldorff M (1997). A spatial scan statistic. Communications in Statistics -Theory and Methods.

[38] Kulldorff M, Feuer EJ, Miller BA, Freedman LS (1997). Breast cancer clusters in the Northeast United States: a geographic analysis. Am J Epidemiology.

[39] Kulldorff Martin, Heffernan Richard, Hartman Jessica, Assunção Renato, Mostashari Farzad (2005). A Space–Time Permutation Scan Statistic for Disease Outbreak Detection. PLoS Medicine.

[40] Cheung YTD, Spittal MJ, Williamson MK, Tung SJ, Pirkis J (2013). Application of scan statistics to detect suicide clusters in Australia. PLoS One.

[41] Coleman M, Coleman M, Mabuza AM, Kok G, Coetzee M, Durrheim DN (2009). Using the SaTScan method to detect local malaria clusters for guiding malaria control programmes. Malar J.

[42] Aamodt Geir, Samuelsen Sven O, Skrondal Anders (2006). International Journal of Health Geographics.

[43] Wand H, Ramjee G (2010). Targeting the hotspots: investigating spatial and demographic variations in HIV infection in small communities in South Africa. J Int AIDS Soc.

[44] Chen J, Roth RE, Naito AT, Lengerich EJ, MacEachren AM (2008). Geovisual analytics to enhance spatial scan statistic interpretation: an analysis of US cervical cancer mortality. Int J Health Geogr.

[45] Zhang W, Wang L, Fang L, Ma J, Xu Y, Jiang J, Hui F, Wang J, Liang S, Yang H (2008). Spatial analysis of malaria in Anhui province, China. Malar J.

[46] Kulldorff M (2002). Commentary: geographical distribution of sporadic Creutzfeldt-Jakob disease in France. Int J Epidemiol.

[47] Xia J, Cai S, Zhang H, Lin W, Fan Y, Qiu J, Sun L, Chang B, Zhang Z, Nie S. Spatial, temporal, and spatiotemporal analysis of malaria in Hubei Province, China from 2004–2011. Malar J. 2015;14(145). 10.1186/s12936-015-0650-2. 10.1186/s12936-015-0650-2 PMC4393858 25879447

[48] Liu Y, Wang X, Liu Y, Sun D, Ding S, Zhang B, Du Z, Xue F (2013). Detecting spatial-temporal clusters of HFMD from 2007 to 2011 in Shandong Province, China. PLoS One.

[49] Bousema T, Griffin JT, Sauerwein RW, Smith DL, Churcher TS, Takken W, Ghani A, Drakeley C, Gosling R (2012). Hitting hotspots: spatial targeting of malaria for control and elimination. PLoS Med.

[50] ESRI (2011). ArcGIS Desktop: Release 10.3.

[51] Vitoria M, Granich R, Gilks CF, Gunneberg C, Hosseini M, Were W, Raviglione M, De Cock KM (2009). The global fight against HIV/AIDS, tuberculosis, and malaria current status and future perspectives. Am J Clin Pathol.

[52] Hotez PJ, Molyneux DH, Fenwick A (2006). Incorporating a rapid-impact package for neglected tropical diseases with programs for HIV/AIDS, tuberculosis and malaria. PLoS Med.

[53] Hotez PJ, Molyneux DH, Fenwick A (2007). Control of neglected tropical diseases. N Engl J Med.

[54] Ngarakana-Gwasira E. T., Bhunu C. P., Masocha M., Mashonjowa E. (2016). Assessing the Role of Climate Change in Malaria Transmission in Africa. Malaria Research and Treatment.

[55] Idemyor V (2007). Human immunodeficiency virus (HIV) and malaria interaction in sub-Saharan Africa: the collision of two titans. HIV Clin Trials.

[56] Korenromp EL, Williams BG, de Vlas SJ, Gouws E, Gilks CF, Ghys PD, Nahlen BL (2005). Malaria attributable to the HIV-1 epidemic sub-Saharan Africa. Emerg Infect Dis.

[57] Cuadros Diego F., Branscum Adam J., García-Ramos Gisela (2011). No Evidence of Association between HIV-1 and Malaria in Populations with Low HIV-1 Prevalence. PLoS ONE.

[58] Ruperez M (2016). Mortality, morbidity, and developmental outcomes in infants born to women who received either Mefloquine or Sulfadoxine-Pyrimethamine as intermittent preventive treatment of malaria in pregnancy: a cohort study. PLoS Med.

[59] Rattanapunya Siwalee, Kuesap Jiraporn, Chaijaroenkul Wanna, Rueangweerayut Ronnatrai, Na-Bangchang Kesara (2015). Prevalence of malaria and HIV coinfection and influence of HIV infection on malaria disease severity in population residing in malaria endemic area along the Thai–Myanmar border. Acta Tropica.

[60] Zimbabwe National Statistics Agency (ZIMSTAT). Zimbabwe Poverty Atlas. Harare, ZIMSTAT; 2015.

[61] Kangoye DT, Noor A, Midega J, Mwongeli J, Mkabili D, Mogeni P, Kerubo C, Akoo P, Mwangangi J, Drakeley C, Marsh K, Bejon P, Njuguna P (2016). Malaria hotspots defined by clinical malaria, asymptomatic carriage, PCR and vector numbers in a low transmission area on the Kenyan coast. Malar J.

[62] Kahn JG, Muraguri N, Harris B, Lugada E, Clasen T, Grabowsky M, Mermin J, Shariff S (2012). Integrated HIV testing, malaria, and Diarrhoea prevention campaign in Kenya: modeled health impact and cost-effectiveness. PLoS One.

[63] Verguet S, Kahn JG, Marseille E, Jiwani A, Kern E, Walson JL (2015). Are long-lasting insecticide-treated bednets and water filters cost-effective tools for delaying HIV disease progression in Kenya?. Glob Health Action.

[64] Adeola AM, Botai OJ, Olwoch JM, Rautenbach CJ, Adisa OM, Taiwo OJ, Kalumba AM (2016). Environmental factors and population at risk of malaria in Nkomazi municipality, South Africa. Trop Med Int Health.

[65] Ayala D, Costantini C, Ose K, Kamdem GC, Antonio-Nkondjio C, Agbor JP, Awono-Ambene P, Fontenille D, Simard F (2009). Habitat suitability and ecological niche profile of major malaria vectors in Cameroon. Malar J.

[66] Rulisa S, Kateera F, Bizimana JP, Agaba S, Dukuzumuremyi J, Baas L (2013). Malaria prevalence, spatial clustering and risk factors in a low endemic area of eastern Rwanda: a cross sectional study. PLoS One.

[67] Sande S, Zimba M, Chinwada P, Masendu HT, Mberikunshe J (2016). Makuwaza a. a review of new challenges and prospects for malaria elimination in Mutare and Mutasa districts, Zimbabwe. Malar J.

[68] Snow R, Guerra C, Noor A, Myint H, Hay S (2005). The global distribution of clinical episodes of plasmodium falciparum malaria. Nature.

